# The effects of *Artemisia aucheri* extract on hepatotoxicity induced by thioacetamide in male rats

**Published:** 2013

**Authors:** Azam Rezaei, Shahnaz ShekarForoush, Saeed Changizi Ashtiyani, Hydar Aqababa, Ali Zarei, Maryam Azizi, Hasan Yarmahmodi

**Affiliations:** 1 *Department of Biology, Arsanjan Branch, Islamic Azad University, Arsanjan, I. R. Iran*; 2 *Department of Physiology, Arak University of Medical Sciences, Arak, I. R. Iran*; 3 *Department of Biology, Damghan Branch, Islamic Azad University, Damghan, I. R. Iran*

**Keywords:** *Artemisia*, Hepato-Protective, Phenolic Compounds, Rat, Thioacetamide

## Abstract

**Objective:** Liver is an important organ that is exposed to many oxidant and carcinogenic agents, thus antioxidant compounds are beneficial for liver health. *Artemisia* contains flavonoid compounds and anti-diabetic, antioxidant, and anti-inflammatory properties. Due to possessing terpene and sesquiterpene compounds, this plant has antioxidant properties. This study was done to investigate the effects of *Artemisia* plant extract on thioacetamide-induced hepatotoxicity in Wistar rats.

**Materials and Methods: **For induction of hepatotoxicity, 50 mg/kg thioacetamide was injected intraperitoneally (i.p). After extraction and purification, the hydroalcoholic extract was injected i.p. at 100, 200, and 300 mg/kg doses for 21 days together with thioacetamide at 50 mg/kg dose in the last 3 days. After blood sampling and separation of serum, alanine aminotransferase (ALT), aspartate aminotransferase (AST), alkaline phosphatase (ALP), albumin, and total protein concentrations were measured.

**Results:** Significant decreases in aminotransferase and alkaline phosphatase activities and significant increases in the concentration of albumin and total protein in groups treated with the extract compared with thioacetamide-treated group were observed (p<0.05).

**Conclusion:** The results indicate that protective effects of *Artemisia* extract against the thioacetamide-induced hepatotoxicity may be due to its ability to block the bioactivation of thioacetamide, primarily by inhibiting the activity of Cyp_450_ and free radicals. *Artemisia* possesses quercetin. Studies have demonstrated that quercetin inhibits lipid peroxidation and as an antioxidant can inhibit lipid peroxidation.

## Introduction

Liver is an important body organ which is responsible for metabolism, bile secretion, and excretion, synthesis, and regulation of essential hormones (Mohammed et al., 2009 ▶). Liver diseases are a major cause of mortality and morbidity in developing countries. Alcoholism, virus-induced chronic liver disease, and using hepatotoxic drugs (antibiotics, carbon tetrachloride, thioacetamide, and acetaminophen) are the major risk factors for liver diseases (Mohammed et al., 2009 ▶; Saleem et al., 2010 ▶; Milagros et al., 2000 ▶). In modern medicine, due to the absence of protective drugs in the treatment of liver diseases, medicinal plants are highly popular (Mohammed et al., 2009 ▶). The history of using herbs in medical practice is a long one (Azadbakht et al., 2003 ▶; Kavita and Sanjay, 2002 ▶). In fact, owing to its lower costs and greater compatibility, herbal medicine has received great attention in recent decades (Azadbakht et al., 2003 ▶). Herbs are rich in different compounds such triglycerides, flavonoids, and polyphenols, that can protect the liver against damages induced by hepatotoxic drugs (Milagros et al., 2000 ▶).

*Dermane and Afsantin* are the names which are applied to the majority of *Artemisia* class of plants which grow in Iran. This class of plants belongs to the *Compositae *family, Radiae (Baluchnejad mojarad et al., 2005 ▶; Mir Heidar, 1994 ▶) that is of different varieties with different names which often have bitter aromatic leaves withmore or less similar medical properties. This plant has 34 varieties with one-year or older herbs scattered throughout Iran, especially its northern regions. The unique varieties of this plant are *Aucheri. A*, *Melanolepis. A*, and *Siberia A* (Mozaffari, 1996 ▶). *Dermane* (*Artemisia*) possesses anti-parasite, anti-bacterial, and anti-diabetic properties (Mozaffari, 1996 ▶; Yalshphe et al., 1979 ▶). It also has antiseptic, anti-cough, anti-flatulent, anti-ascaris, anti-febrile, appetizing, and anti-inflammatory properties and can be applied to the treatment of headache and abdominal pains. In the past, this plant was used as an analgesic in sedating neuralgia and treatment of hepatitis (Baluchnejad mojarad et al., 2005 ▶; Mir Heidar, 1994 ▶, Dashti et al., 2011 ▶; Twaij and Al-Badr, 1988 ▶). 

*Artemisia* has anti-inflammatory (Dashti et al., 2011 ▶, Sadeghifard and Zareian, 2009 ▶; Tignox and Gumila, 2000 ▶), anti-tumor (Kim et al., 2004 ▶; Emami et al., 2010 ▶), anti-stomach ulcer (Emami et al., 2010 ▶; Foglio et al., 2002 ▶), and antioxidant (Cordova et al., 2002; Kim et al., 2003 ▶) effects. This plant contains various flavonoids including quercetin and retinoid (Bahrami-Karkevandi et al., 2003 ▶; Asgari et al., 2008a ▶, Farzaneh et al., 2006 ▶) and most of its varieties have colorgenic, sesquiterpene, and monoterpenes (Jafari Dinani et al., 2007 ▶; Soon et al., 1997 ▶) which have strong antioxidant properties. To date, no studies have been done on the impact of *Artemisia* on hepatotoxicity induced by thioacetamide, hence the present study was conducted to investigate the effects of *Artemisia* plant extract on thioacetamide-induced liver toxicity.

**Figure 1:**
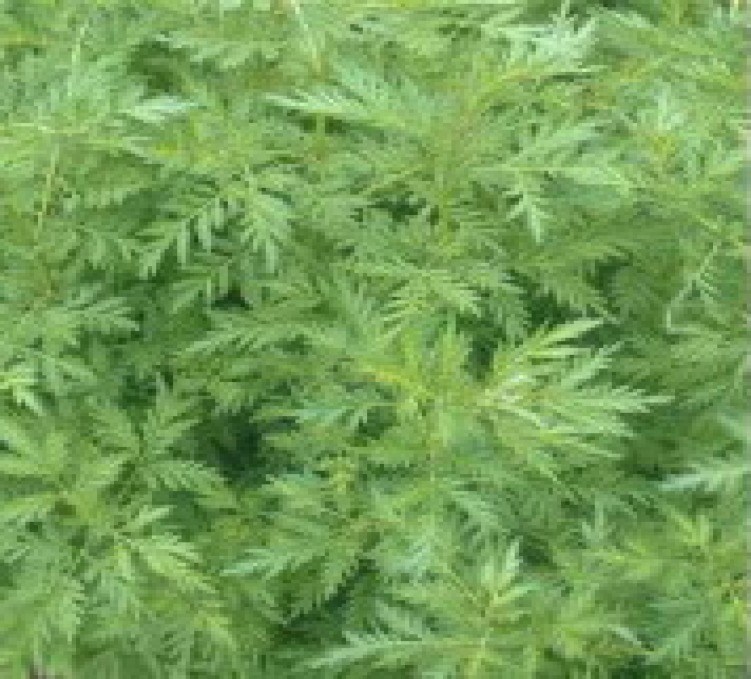
*Artemisia Aucheri *leaves

## Materials and Methods

This experimental study was done using 48 male Wistar rats supplied from Tehran University of Medical Sciences that were transferred to Animal Breeding Center of Azad University of Arsanjan University. Animal care and handling were performed according to the guidelines set by Iranian Ministry of Health and Medical Education. The animals were placed in controlled conditions of temperature (25±2 ^°^C) and light (light: 12 hours, dark: 12 hours). Before launching the experiment, the rats were weighed so that they were within a specific weight range (190±5 g). They were provided with standard food and water ad libitum. The rats aged 2.5-3 months old and were randomly divided into six groups (n= 8 for each group):

Control group: Throughout the experiment, the rats did not receive any vehicle or drugs. Sham group: The rats were administered 0.5 ml of the solvent (distilled water) intraperitoneally. Thioacetamide group: In this group, 50 mg/kg of** thioacetamide **was intraperitoneally administered to the rats. Treatment group 1: The rats were intraperitoneally administered 100 mg/kg of the alcoholic extract of *Artemisia* together with 50 mg/kg of thioacetamide*,* in the last three days, over 21 days. Treatment group 2: The rats were intraperitoneally administered 200 mg/kg of the alcoholic extract of *Artemisia* together with 50 mg/kg of thioacetamide, in the last three days, over 21 days. Treatment group 3: The rats were intraperitoneally administered 300 mg/kg of the alcoholic extract of *Artemisia* together with 50 mg/kg of thioacetamide, in the last three days, over 21 days.

**Preparation of the Extract**

After drying, the flowered branches of *Artemisia* were powdered. Next, 1000 g of the resulting powder was poured into an Erlenmeyer flask and 400 cc of 90% ethylic alcohol was added to it so that the powder was covered by the alcohol. After 24 hours, the mixture was placed on the shaker and the obtained extract was filtered by filtering paper and a funnel. Afterward, the remaining solid was mixed with ethylic alcohol 70% and placed on the shaker for 24 hours. The obtained extract was filtered, refined, and added to the first extract. Then, the extract was distilled in distiller device at vacuum at 60 ^°^C and 70 rpm until the remaining volume decreased to one fifth of the primary volume. The extract container was then removed from the device and the remaining.

 The **extract** then **decanted three times** by **50** ml of **chloroform.** The remaining was poured into pottery container and was left to be dried at 50 ^°^C in oven. The average dried extract obtained through this method was 68 g per kilogram plant powder (the herbarium code of the plant is 14067) (Bahrami-Karkevandi et al., 2011 ▶).

**Data Analysis**

The obtained mean values for the liver enzymes and concentrations in different groups were statistically analyzed by ANOVA and Tukey’s test. F-test was run to determine the degree of significance (p<0.05). Enzymes, i.e., alanine aminotransferase (ALT), alkaline phosphatase (ALP), aspartate aminotransferase (AST), total protein, and albumin were assessed using radio immunoassay (RIA) and pars Azmoon kit using RIA1000 (USA). All obtained values are expressed us meant±SD and data analyses were done using SPSS ver. 11.5. 

**Blood Sampling**

Forty eight hours after the last injection, the rats were anesthetized with chloroform and blood samples were drawn directly from their hearts. Serum was separated by centrifuging. Serum transaminase and total albumin and protein were measured for assaying and making intergroup comparisons of liver function.

## Results

Comparison of the results of studies on Artemisia extract together with thioacetamide on the activity level of liver enzymes shows that in terms of ALT, there was no significant difference between the sham group and control group.In the group receiving just thioacetamide, there was a significant increase in ALT compared with the sham group (p<0.05). Moreover, there was a significant decrease in ALT in experimental groups that received Artemisia extract together with thioacetamide compared with the group that received just thioacetamide (p<0.05).

ALT decreased dose dependently to normal value by the extract (p<0.05). There was no significant difference between experimental groups that received Artemisia extract (p<0.05). In terms of ALP, the group receiving thioacetamide only showed significant increase compared with the sham group (p<0.05). Moreover, there was a significant decrease in ALP in experimental groups that received Artemisia extract together with thioacetamide compared with the group that received just thioacetamide (p<0.05).

In the experimental group that received Artemisia extract at dosage of 100 mg/kg, there was a significant increase in ALP compared with the sham group (p<0.05). There was also a significant decrease in ALP in experimental groups that received Artemisia extract at 100 mg/kg compared with those which received 200 mg/kg of the plant, and the group that received 100 mg/kg compared with the group that received 300 mg/kg of the extract (p<0.05).

In terms of AST, the group that received thioacetamide showed only significant increase compared with the sham group (p<0.05). Moreover, there was a significant decrease in AST in experimental groups that received Artemisia extract together with thioacetamide compared with the group that received just thioacetamide (p<0.05).

In the experimental groups that received Artemisia extract at dosage of 100-200 mg/kg, there was a significant decrease in AST (p<0.05).

In the group that received Artemisia extract, there was a significant decrease in AST compared with the sham group (p<0.05). There was a significant increase in albumin in the group that received Artemisia extract together with thioacetamide compared with the group that received just thioacetamide (p<0.05). In the experimental groups that received Artemisia extract at dosage of 100 mg/kg, there was a significant decrease in albumin compared with the sham group (p<0.05). In the experimental groups that received Artemisia extract, there was no significant difference in the albumin level (p<0.05).

In terms of total protein, there was no significant difference between the control group and sham group. The group that received thioacetamide only showed significant decrease in total protein compared with the sham group (p<0.05). There was a significant increase in the total protein in the group that received Artemisia extract together with thioacetamide compared with the group that received just thioacetamide (p<0.05). There was no significant difference between the experimental groups and sham group (p<0.05). In terms of total protein, there was also no significant difference between the experimental groups that received Artemisia extract (Table 1).

**Table 1:** The Effects of *Artemisia Aucheri* Extract (AE) in different doses on serum concentrations of protein, alanine aminotransferase (ALT), aspartate aminotransferase (AST), and alkaline phosphatase (ALP) after hepatotoxicity induced by thioacetamide

**Parameters **	**ALT**	**AST**	**ALP**	**Albumin**	**Protein**
**Group**					
**Control**	7.7±0.1	4.1±0.2	540.1±3.4	183.5±3.3	63.9±1.3
**Sham**	7.8±0.1	4.3±0.2	573±8.9	207.8±2.2	68.6±1.7
**Thioacetamide**	7.3±0.1#	3.±0.2#	573.3±8.9 #	409.3±4.5 #	107.3±3.7 #
***AE*** ** (100mg/Kg) **	7.9±0.3*	3.6±0.2#*	738.7±12.6#*	329.7±25 #*	82±6.2 #*
***AE*** ** (200mg/Kg)**	8.00.1 *	3.8±0.05#†*	606±11.4 #†*	267.9±10 #†*	75.4±4.1*
***AE (*** **300mg/Kg** ***)***	7.8±0.2 *	3.9±0.07 *	602.6±5.5 #†*	276.6±14.4#*	68.9±4.2*

* = indicates significant changes compared to the thioacetamide group (p<0.05);

# = indicates significant changes compared to the Sham group (p<0.05);

† = comparison between minimum and maximum dose (p<0.05)

## Discussion

The results of this experiment showed that the amount of AST, ALT, and ALP enzymes activity in the group treated with thioacetamide presented a significant increase in comparison with the sham group. Concomitant injection of *Artemisia* plant extract and thioacetamide resulted in the reduction of AST, ALP, and ALT activities compared with the thioacetamide group. In addition, the obtained results indicate that the albumin and plasma proteins in the treatment group that received thioacetamide significantly decreased compared with the sham group, whereas concomitant injection of *Artemisia* plant extract and thioacetamide led to a significant increase in albumin and plasma protein values compared with the group that received thioacetamide alone. Thioacetamide is a known hepatotoxin that induces liver necrosis by producing free radicals (Mohammed et al., 2009 ▶; Madani et al., 2006b ▶; Sun et al., 2000 ▶). In a short period of time, thioacetamide induces liver damages (Minnady et al., 2010 ▶). This toxin is metabolized by detoxification enzymes of P450 cytochrome system (Mohammed et al., 2009 ▶; Madani et al., 2006b ▶). 

Thioacetamide metabolites result in the production of thioacetamide S-oxide that attacks membrane proteins and lipids, changes the cell permanently, and increases intracellular calcium concentrations through increasing the volume of nucleus (Mohammed et al., 2009 ▶; Minnady et al., 2010 ▶; Madani et al., 2006a ▶). Moreover, thioacetamide causes the inhibition of mitochondria and eventually liver necrosis (Minnady et al., 2010 ▶). Increased liver enzymes, AST, and ALT are good indicators of liver necrosis (Mohammed et al., 2009 ▶; Minnady et al., 2010 ▶). Cellular damages are identified by increases in serum ALP, ALT, and AST levels in that these enzymes are in the cytoplasm and after cellular damage they enter blood circulation (Mohammed et al., 2009 ▶; Madani et al., 2006b ▶; Minnady et al., 2010 ▶). Zaragoza et al. (2005) ▶ reported that thioacetamide leads to AST enzyme increase (Zaragoza et al., 2005 ▶). Bassi (2004) ▶ stated that thioacetamide increases ALT and AST enzymes concentrations (Bassi et al., 2004 ▶). Free radicals damage cell membranes such as hepatocytes which results in increasing liver enzymes activity. 

This causes the liver enzymes which are normally located inside cell cytosols to enter blood circulation. Increased activity of these enzymes indicates the degree and type of liver damage (Shariati and Zarei, 2006 ▶; Taheri et al., 2012 ▶). Phenolic antioxidant compounds are capable of damaging free radicals in the body (Mohammed et al., 2009 ▶). Cordova et al.’s study (2002) showed that polyphenolic compounds, especially flavonoids, initially have an inhibitory effect on cytochrome P_450_ system and prevent the further metabolism of thioacetamide which eventually lead to the reduction of free radicals production (Cordova et al., 2002). These compounds, due to their antioxidant property, are capable of neutralizing free radicals present in the cell environment and prevent their damaging effects (Madani et al., 2006 a ▶, 2006 b ▶; Sun et al., 2000 ▶; Pyo et al., 2004 ▶). Studies done on flavonoids compounds have demonstrated that these compounds can revive cells against glutathione depletion and by increasing the capacity of antioxidant enzymes (glutathione, glutathione reductase, glutathione peroxidase, and catalase) protect them (Bahrami-Karkevandi et al., 2003 ▶; Baer-Dubowska et al., 1998 ▶). *Artemisia* possesses hepato-protective properties due to its reviving capability and its potential for neutralizing free radicals (Janbaz and Gilani, 1995 ▶). *Artemisia* extract prevents lipid peroxidation and increases the activity of antioxidant enzymes (Kim et al., 2003 ▶; Jafari Dinani et al., 2007 ▶). *Artemisia* plant extract has also shown to be capable of trapping oxygen free radicals. *Artemisia* plant extract is replete with cholinergic acid which has an anti-free radical activity that destroys oxygen free radicals (Mirzaei et al., 2010 ▶, Soon et al., 1997 ▶). It also inhibits **heme oxygenase**; thus, it reduces the production of free radicals (Jafari Dinani et al., 2007 ▶; Pan, 2003). Studies have demonstrated that quercetin inhibits lipid peroxidation (Romaiana et al., 2009 ▶) and as an **antioxidant**, it has a major role against free radicals in various diseases, including liver diseases. The chief mechanism of this substance is protecting cells against oxidative radicals (Bilyk and Sapres, 1985 ▶). Studies done by Madanni *et al*. on *Cichorium intybus L*, *Silybum marianum*, and *Calendula officinalis* plants have revealed that these plants, due to possessing flavonoid compounds, protect cells against the oxidative effects of thioacetamide (Madani, 2006a ▶, 2006b ▶; Asgari et al., 2005b ▶). This protective effect of *Artemisia* plant extract is probably due to the presence of flavonoids compounds and sesquiterpenes in it. Minnaday’s study on the effect of oyster mushroom (*Pleurotus Florida*) on hepatotoxicity induced by thioacetamide indicated that *Pleurotus Florida* extract due to possessing antioxidant compounds has a significant effect on decreasing the level of liver enzymes and increasing total body protein (Minnady et al., 2010 ▶). 

Alkaline phosphatase is a transpeptidase that increases in bone and liver diseases. Several studies have shown that phenolic compounds present in medicinal plants can prevent the toxic effects on the liver and result in reduction of glutamic pyruvic transaminase and alkaline phosphatase release into blood (Taheri et al., 2012 ▶). Reduction of liver cells damage leads to the reduction in the level of ALT and AST enzymes in plasma (Madani et al., 2009c ▶). 
